# Forebrain-Specific Loss of BMPRII in Mice Reduces Anxiety and Increases Object Exploration

**DOI:** 10.1371/journal.pone.0139860

**Published:** 2015-10-07

**Authors:** Zofeyah L. McBrayer, Jiva Dimova, Marc T. Pisansky, Mu Sun, Hideyuki Beppu, Jonathan C. Gewirtz, Michael B. O’Connor

**Affiliations:** 1 Department of Genetics, Cell Biology, and Development, University of Minnesota, Minneapolis, Minnesota, United States of America; 2 Department of Psychology and Neuroscience, University of Minnesota, Minneapolis, Minnesota, United States of America; 3 Department of Clinical and Molecular Pathology, University of Toyama, Toyama, Japan; Columbia University, UNITED STATES

## Abstract

To investigate the role of Bone Morphogenic Protein Receptor Type II (BMPRII) in learning, memory, and exploratory behavior in mice, a tissue-specific knockout of BMPRII in the post-natal hippocampus and forebrain was generated. We found that BMPRII mutant mice had normal spatial learning and memory in the Morris water maze, but showed significantly reduced swimming speeds with increased floating behavior. Further analysis using the Porsolt Swim Test to investigate behavioral despair did not reveal any differences in immobility between mutants and controls. In the Elevated Plus Maze, BMPRII mutants and Smad4 mutants showed reduced anxiety, while in exploratory tests, BMPRII mutants showed more interest in object exploration. These results suggest that loss of BMPRII in the mouse hippocampus and forebrain does not disrupt spatial learning and memory encoding, but instead impacts exploratory and anxiety-related behaviors.

## Introduction

Bone Morphogenic Proteins (BMPs) form the largest subclass of secreted signaling factors within the TGF-β (Transforming Growth Factor) superfamily. These factors control a plethora of developmental and homostatic processes in higher eurkayotes including, axial development, tissue patterning, wound healing, immune regulation and apoptotic responses. BMP ligands form homeomeric or heteromeric dimers that bind to type I (BMPRIA or BMPRIB) and type II (BMPRII, ACTRII, and ACTRIIB) receptors. Ligand binding stabilizes the interaction between type I and type II receptors, which allows phosphorylation of the GS domain in the type I receptor by the constitutively active kinase domain of the type II receptor. Phosphorylation of the GS domain provides a substrate for the recruitment of Smad proteins, which are the major transducers of signaling. Smads 1/5/8 are the BMP-specific regulatory Smads (R-Smads). After phosphorylation by the activated receptor complex, the activated R-Smads partner with a common Smad (Smad 4), and the stabilized complex accumulates in the nucleus where it mediates transcriptional outputs in collaboration with other co-activators or co-repressors [1–3]

BMP signaling is involved in many aspects of embryonic development, including the formation and patterning of the central and peripheral nervous system [4,5]. After embryonic development, many components of the BMP signaling pathway continue to be expressed in the rodent brain, and several reports indicate that there is high expression of BMP signaling components in the adult rodent hippocampus [6–15]. The hippocampus is important for acquisition of declarative memories in humans and for spatial memory in animal models [16,17]. Also, previous mouse loss-of-function studies in which extracellular BMP inhibitors, BMPRI, or Smad proteins where specifcally removed from the forebrain showed alterations in learning, as well as anxiety-like, fear-related, or exploratory behavior [18–21]. Furthermore, studies of mice that overexpress BMP ligands or various extracellular BMP inhibitors in the brain also support a role for BMP signaling in modulating learning, memory, and cognition [22,23].

BMPRII is highly expressed in the CA1-3 regions of the hippocampus and in the dentate gyrus, consistent with a potential role in learning, memory, and cognition. However, conventional loss of BMPRII is embryonic lethal at gastrulation [24]. Therefore, in order to examine the explicit contribution of BMPRII to learning and memory in adult mice, we used the promoter of calcium/calmodulin-dependent protein kinase II-α to drive expression of Cre-Recombinase (CaMKIIα-Cre) in a BMPRRII floxed mouse resulting in the specific removal of BMPRII in the postnatal hippocampus and forebrain [25–27]. The learning, memory, anxiety-related, and exploratory behavior of these conditional mutant mice and their littermate controls were examined using the Elevated Plus Maze (EPM), the object exploration test, the Morris water maze (MWM), the Porsolt Swim test (PST), and prepulse inhibition (PPI). The results of these tests suggest that loss of BMPRII in the mouse hippocampus and forebrain does not disrupt spatial learning and memory, but does affect exploratory and anxiety-related behaviors.

## Materials and Methods

### Mouse Strains and Breeding

Mice were bred onto a C57BL6/J background. The 2loxP conditional BMPRII (BMPRII floxed) mouse strain was a kind gift of the En Li lab at Harvard Medical School [25]. The 2loxP conditional allele has the pgk-neo cassette removed and has loxP sites flanking exons 4 and 5 of BMPRII. Exons 4 and 5 encode the transmembrane domain and a portion of the kinase domain of BMPRII. The Smad4 conditional (Smad4 floxed) mouse strain has loxP sites flanking the eighth exon of Smad4 for excision by Cre-Recombinase [28–30].

The calcium/calmodulin-dependent protein kinase II-α driven Cre-Recombinase (CaMKIIα-Cre) mouse lines were a kind gift from Ioannis Dragatsis and Scott Zeitlin at Columbia University [26,27]. The BMPRII floxed line carries the L7ag#13 allele of CamKIIα-Cre, which expresses predominantly in the CA1-3 regions of the hippocampus and in the dentate gyrus. The Smad4 floxed line carries the R1Ag5 allele of CaMKIIα-Cre, which expresses predominantly in the CA1 region of the hippocampus and in the dentate gyrus. Both lines are reported to have high expression levels in all forebrain structures and moderate levels in the cerebellum. The highest level of transgene expression and recombination in neuronal cells is detected at P5. Transgene expression is also detected in the testes in both lines.

The BMPRII mutant line was maintained by crossing BMPRII ^flox/flox^; + females to BMPRII ^flox/flox^; CaMKIIα-Cre males (single copy of Cre). Although the cross could be set in either direction by 8 weeks of age, BMPRII ^flox/flox^; CaMKIIα-Cre males crossed to BMPRII ^flox/flox^; + females often stopped producing after 1–3 litters and males 4 months or older often produced no litters, but BMPRII ^flox/flox^; + females were usually good mothers. Conversely, BMPRII ^flox/flox^; CaMKIIα-Cre females had a greater tendency to cannibalize the pups. Use of Mouse Igloos (Bio-Serv) and supplementation with chocolate Mini treats (Bio-Serv) from day 14 of pregnancy until pups had fur helped increase pup survival by decreasing cannibalism. Cage cleaning during the first week after birth was also avoided. The establishment of the Smad4^flox/flox^; CaMKIIα-Cre line is previously described [18]. The line was maintained by crossing Smad4^flox/flox^; CaMKIIα-Cre females to Smad4^flox/flox^; + males due to poor breeding using Smad4^flox/flox^; CaMKIIα-Cre males. In contrast to BMPRII mutant females, Smad4 mutant females were usually good mothers, and often continued to produce litters even after six months of age.

### Genotyping

The genotypes of mice were identified by polymerase chain reaction (PCR) of DNA extracted from tail tissue. BMPRII floxed mice were identified using primer sequences previously described: BMPRII Primer A (5’-CACACCAGCCTTATACTCTAGATA C–3’) and BMPRII Primer 6R (5’-CACATATCTGTTATGAAACTTGAG–3’) [25]. Smad4 floxed mice were identified using primer sequences previously described [29]: primer Smad4-9 (5’-GGG CAG CGT AGC ATA TAA GA–3’) and primer Smad4-10 (GAC CCA AAC GTC ACC TTC AC–3’)—erratum Yang et al, 2002. CaMKIIα-Cre positive mice were identified using the following sequences: Cre-F (5’-CTG CCACGACCAAGTGACAGC–3’) and Cre-R (5’-CTTCTCTACACCTGCGGTGCT–3’).

### Western Blot Analysis

For Western blot analysis, whole hippocampi and samples of the cerebellum and cortex were dissected. Tissues were lysed in modified RIPA buffer containing 20mM Tris-HCl pH8.0, 150mM NaCl, 1% NP–40/IGEPAL, 1mM EDTA, and 1 Complete Mini protease inhibitor tablet (Roche). BMPRII protein expression was analyzed by standard SDS PAGE using a 7.5% separation gel (pH 8.8) and a 5% stacking gel (pH 6.8). Proteins were transferred onto a PVDF membrane by semi-dry transfer and membranes were blocked in TBS with 0.1% Tween 20 and 5% non-fat milk protein for 1 hour at room temperature. Primary antibodies were incubated overnight at 4°C in blocking buffer. Monoclonal mouse anti-BMPRII (BD Transduction Laboratories) was used at 1/250 and polyclonal rabbit anti-GAPDH (Imgenex) was used at 1/1000. Secondary antibodies were incubated for 1 hour at room temperature in blocking buffer. Monoclonal mouse anti-rabbit HRP and donkey anti-mouse HPR (Jackson Laboratories) were used at 1/100,000. Protein expression was detected by chemiluminescence (Pierce ECL2 Western Blotting Substrate).

### Behavioral Analyses

All procedures involving mice were carried out in accordance with the recommendations of the National Institutes of Health Guide for the Care and Use of Laboratory Animals, and all protocols were reviewed and approved by the Institutional Animal Care and Use Committee (IACUC) at the University of Minnesota.

Male BMPRII ^flox/flox^; CamKIIα-Cre and BMPRII ^flox/flox^; + control littermates were used for behavioral experiments at 2–4 months of age. Mice were housed in groups of 2–4 littermates per cage at weaning. Group 1: naïve mice were first tested in the object exploration assay and then subsequently used in the MWM task, followed by testing for acoustic startle and PPI. Group 2: naïve mice were tested in the EPM and then the PST. Group 3: naïve Smad4 mice were tested in the EPM only. Results of other behavioral tests are previously described [18].

Behavioral tests were conducted in a designated behavior room in order to provide a suitably quiet environment and the necessary equipment for behavioral assays. The room was set to a 12-hr light:dark cycle and the temperature was maintained at 24±2°C. Mice were moved from a general mouse facility into the room at least 2–3 days before testing for acclimation, and housed there for the duration of testing. During this acclimation period, the mice were also handled by the experimenter to prepare them for handling during testing. All testing was performed during the light phase of the light:dark cycle.

### Object Exploratory Test

A single mouse was placed inside a 50cm x 50cm x 40cm (LWH) plastic box with a 4cm round bottle cap (novel object) in one corner, and allowed to roam freely for 15 minutes. Up to 4 boxes could be recorded at a time. Boxes were arranged in a 2X2 configuration that was surrounded by curtains on all sides to reduce the influence of outside cues and distractions. All mice started the experiment at the same time by placing the mice inside a plastic cylinder. The handler then simultaneously lifted all four cylinders via a lever and cord. All sniffing events were validated by eye to exclude false events detected by the software. The boxes used for this experiment were washed using a 3% bleach and detergent solution. Objects were washed with soap and water, rinsed with clean water, and dried between mice to remove odor cues.

### Elevated Plus Maze (EPM)

For the EPM, mice started in the closed arm and were allowed to explore for 10 min. The light level was 200–250 lux for BMPRII mice and 50–60 lux for Smad4 mice. Between trails, the maze was cleaned with a mild 3% bleach and detergent solution to remove urine and odor cues. Open and closed arm exploration was measured as follows: (open arm time/total arm time vs. closed arm time/total arm time). The center zone connecting all arms was excluded.

The EPM apparatus was from Med Associates Inc. The arm dimensions were 34cm x 7cm (length x width), and the wall height of the closed arms was 19cm made from solid black plastic. The stand height of the maze was 90cm. Video footage was captured using a Fire-i^TM^ digital camera from Unibran Co. mounted onto the overhead bar accessory available from Med Associates Inc. for the EPM. An additional metal bar was installed cross-wise to the overhead bar to provide illumination over the open arms. Each end had a halogen light affixed, whose intensity could be manually set by a dimmer switch. Lighting on the arms was measured using a light meter.

### Porsolt Swim Test (PST)

For the PST, mice were placed in the water and allowed to swim for 6 min total. Video footage was captured in AnyMaze^TM^ using a USB digital camera. Immobility was scored manually from 2–6 min, the first two minutes were not scored. Latency to first mobility was scored starting from the time the mouse entered the water. After swimming, the mice were returned to the home cage, which was filled with crushed paper towels to aid in drying. Additionally, a heating pad was placed beneath one side of the cage for warmth.

The PST apparatus was a clear glass container ordered from a vase company. The height was 25cm and the diameter was 20cm. During testing, the water level was filled about 12-13cm high so mice could not touch the bottom. The water was kept at room temperature (approximately 25°C), and was changed after each mouse to remove urine odors and feces floating in the water.

### Morris Water Maze (MWM) Analysis

The MWM was separated into three phases: the cued phase (Day 1), the first hidden phase (Days 2–10) and probe trial (Day 11), and the reversal phase (Days 12–16) and reverse probe trial (Day 17). On the cued day the platform (15cm) was above water and cued with a red flag (SW quadrant). Prior to swimming, mice were placed on the platform and allowed to explore for 25 seconds for both trials. Then they were picked up, taken to a designated release position along the wall, and gently released into the water facing the wall. For the hidden phase, a smaller platform (10cm) was placed in a new quadrant (NW) and submerged 0.5 cm below water. On these days, mice were not given prior exposure to the platform location and were instead taken directly to the designated release position and lowered into the water facing the wall. No platform was present on the probe trial days. For the reversal phase, the platform (10cm) was moved to a new location (SE quadrant). Mice swam two trials per day with an interval of 25–30 min between trials, except on probe trial days, in which mice swam once for 60 s. Mice were allowed to swim for a maximum of 90 s during trials and wait on the platform for 15–20 s. If they did not find the platform, they were gently guided by hand. After swimming, mice were returned to their cage, containing paper towels for drying. All towels were removed after the final swimming trials of the day were finished. Additionally, mice were always provided with sufficient nesting material in their cages for warmth. The pool was 1.2m in diameter and 70cm high. The water temperature was maintained at 24.5±0.5°C. Non-toxic white Tempera® paint was added to the pool to obscure the water for hidden platform trials. Mice were put into groups of 8, 4 mutants and 4 controls. Four release positions were chosen based on the cardinal directions of the pool. When the cued platform was in the SW quadrant, the starting positions were NW, N, E, and SE. When the hidden platform was in the NW quadrant, the starting positions were NE, E, S, and SW. During the reversal test the platform was hidden in the SE quadrant, and the starting positions were NE, N W, and SW. The position directly across from the platform was only used on the probe trial days for all the mice **(S1 Fig)**. Positions were distributed such that each position was used four times per day and no mouse could swim from the same position twice. By the end of 10 days, each mouse started from each spot evenly. Since the reversal only had 5 days, on the 5^th^ day mice started from a position on one side of the platform for trial 1, and started from a position on the other side of the platform for trial 2.

### Acoustic Startle Response

Prepulse inhibition (PPI) was measured using the MedAssociates Inc. Startle Reflex System (St. Albans, VT, USA). The apparatus consisted of four identical Plexiglas cages attached to a load cell platform (PHM–250). Each cage was located in a ventilated, sound-attenuated chamber. The ventilation fans elevated background noise to 65 dB. Cage movements resulted in the displacement of the load cell and voltage signals were amplified and digitized on a scale of arbitrary units by an analog-to-digital converter (ANL-925C Amplifier). Startle amplitude was defined as the peak voltage that occurred during the first 300 msec after onset of a startle stimulus. High-frequency speakers (range 5 to 40 kHz) located 4 cm behind each cage delivered the startle stimuli, which consisted of 40 msec bursts of white noise (22kHz, 3 msec rise time). Noise burst stimuli were of either low intensity i.e., 70, 77, and 83 dB (stimuli defined as prepulses) or of high intensity i.e., 100, 110, and 120 dB (stimuli defined as pulses).

Both mutant and control mice received a single day of testing. All animals underwent a brief (5-min) acclimation period immediately after they were introduced to the experimental apparatus. Test trials consisted of a pulse stimulus, prepulse stimulus, or a combination of pulse and prepulse. On the latter trial type, all prepulse stimuli were presented 120 msec prior to the pulse stimulus. Each type of trial was presented 10 times with variable ISI (average 20 seconds, range 15–25 seconds). Prepulse inhibition was defined as absolute percent change in startle when the prepulse preceded the pulse, i.e., (pulse alone trials-prepulse and pulse trials)/pulse alone trials. In order to detect differences in locomotor activity between mutant and control mice, the animal’s activity was also recorded in the absence of any startle stimuli.

### Software

Object exploratory behavior and swimming behavior was captured using an overhead video camera, and the data was analyzed using the TopScan® software from Clever Systems Incorporated. The EPM and FST video footage was captured in AnyMaze^TM^ using a USB digital camera. Open and closed arm events in the EPM were analyzed in AnyMaze^TM^, while immobility and latency to immobility in the PST was manually scored for accuracy.

### Statistical Analysis

Unpaired t-tests were used to compare genotype effects for all behavioral tests. For PPI data, additional three-way ANOVAs were used to analyze interactions between genotype, prepulse levels, and pulse levels. Genotype served as the between group variable; prepulse or pulse intensity acted as the within group, repeated measure. For the hidden and reversal phases of the MWM and object exploration over time, a two-way ANOVA with repeated measures was performed, and Bonferroni’s adjustment for multiple hypothesis testing was used during *post hoc* analysis. For all statistical analyses, significance was determined as alpha levels less than 0.05.

## Results

### Reduced BMPRII Protein Expression in the Hippocampus and Forebrain of fbΔBMPRII Mice

**Fig 1:**
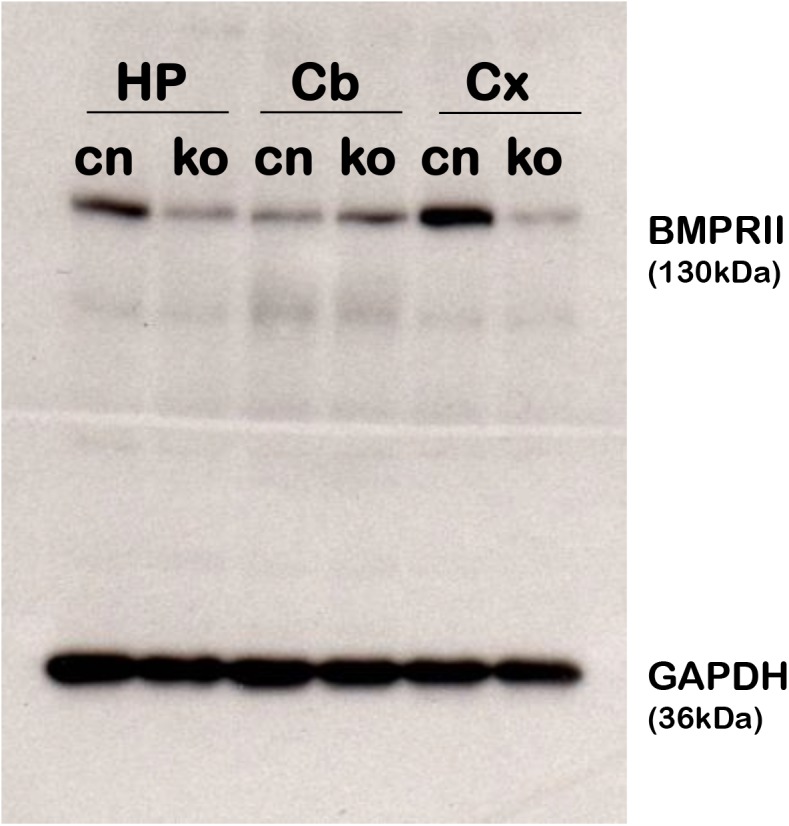
Western Blot Analysis of BMPRII Protein in the Brain. Western blot analysis of tissues from the hippocampus (HP), Cerebellum (Cb), and cortex (Cx) in BMPRII flox/flox controls (cn) and BMPRII flox/flox; CaMKIIα-Cre (ko) mice. At 2 months old, fbΔBMPRII mutant mice show a great reduction of BMPRII protein in the hippocampus and cortex, but show similar levels of BMPRII protein in the cerebellum.

In order to investigate the role of BMP signaling in learning and memory, we generated a tissue-specific knockout of BMPRII in the postnatal hippocampus and forebrain using the L7ag#13 line of CaMKIIα-Cre to delete a floxed allele of BMPRII [25] beginning at postnatal day 5 [26,27]. These mice will be referred to henceforth as fbΔBMPRII mice. In order to determine the efficacy of the deletion, we measured BMPRII protein levels by Western blot analysis. Whole hippocampi and samples of the cerebellum and cortex were dissected from fbΔBMPRII mice and control littermates (BMPRII^flox/flox^; +). Western blot analysis revealed that at 2 months of age, fbΔBMPRII mice had a significant reduction of BMPRII protein in the hippocampus and cortex, compared to control littermates, but similar levels of protein in the cerebellum **(Fig 1).** The residual BMPRII protein detected in mutant mice likely reflects expression in astrocytes and other glia within the hippocampus that do not express Cre using the CaMKIIα driver [18].

### The fbΔBMPRII Mice Show Reduced Anxiety-Like Behavior in the Elevated Plus Maze

Previous studies have suggested a role for BMP signaling in the modulation of anxiety-related behavior in rodents. Mice doubly mutant for BMPRIA and BMPRIB in the forebrain produced using EMX1-(IRES)-Cre show reduced anxiety-like behavior [21]. Therefore we investigated whether signaling specifically through BMPRII contributes to this behavior.

In order to study anxiety-related behavior, we used the Elevated Plus Maze (EPM), which is a standard test for evaluating anxiety-related behavior in mice [31–34]. Anxiety-related behavior is assessed by analyzing the time spent in the “open” unprotected arms and the protected “closed” arms of the maze. Reduced anxiety-like behavior is correlated with increased open-arm exploration upon application of anxiolytic drugs, while increased anxiety-like behavior is correlated with reduced open-arm exploration and increased closed-arm time, upon application anxiogenic drugs [31].

Before testing, small preliminary trials were run to determine an appropriate light level. Final testing was carried out at approximately 200-250lux since the higher light intensity is expected to promote more anxiety.

**Fig 2:**
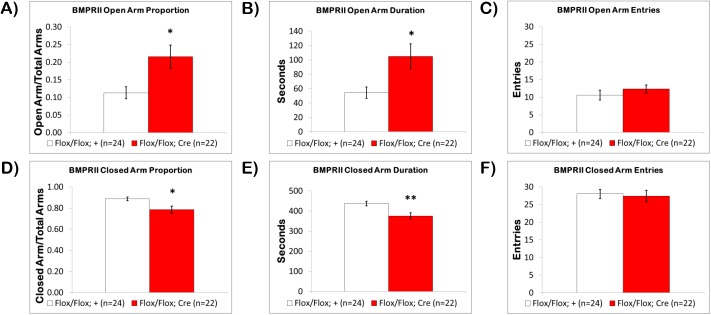
Loss of BMPRII Modulates Anxiety-Related Behavior in the Elevated Plus Maze. In the Elevated Plus Maze (A) the fbΔBMPRII mutant mice (red) spent a significantly increased proportion of time exploring the open-arms compared to control littermates (white), and (B) had a significantly increased duration of time on the open-arms, (C) but there was no significant difference in open-arm entries. When closed-arm exploration was examined, (D) the fbΔBMPRII mutant mice spent a significantly reduced proportion of time on the closed-arms, and (E) had a significantly reduced duration of time on the closed-arms, (F) but there was no significant difference in closed-arm entries. Results reported as mean ± S.E.M. Asterisk indicates *p<0.05 or **p<0.005.

In this task, naïve fbΔBMPRII mutants spent significantly more time exploring the open-arms. The proportion of time spent on the open-arms **(Fig 2A)** for fbΔBMPRII mice was 21.6±3.3% compared to 11.3±1.7% for control littermates (p<0.01). The duration of time on the open-arms was also significantly different (p<0.01), and nearly double, with fbΔBMPRII mice spending 104.9±17.5 seconds compared to 54.5±7.9 seconds in control littermates **(Fig 2B)**. No significant differences in open-arm entries were seen **(Fig 2C)**.

When closed-arm time was examined, naïve fbΔBMPRII mice spent significantly less time in the closed-arms. The proportion of time spent in the closed-arms **(Fig 2D)** for fbΔBMPRII mice was 78.4±3.3% compared to 88.7±1.7% for control littermates (p<0.01). The duration of time in the closed-arms was also significantly reduced (p<0.005) with fbΔBMPRII mice spending 375.0±15.8 seconds compared to 437.6±12.0 seconds in control littermates **(Fig 2E)**. Lastly, there were no differences in closed-arm entries between fbΔBMPRII mice and control littermates **(Fig 2F)**. These results indicate that fbΔBMPRII mice show reduced anxiety-like behavior and suggest a role for BMP signaling through BMPRII in the modulation of anxiety-related behaviors in mice.

### The fbΔSmad4 mice Show Reduced Anxiety-Like Behavior in the Elevated Plus Maze

The reduced anxiety-like behavior in fbΔBMPRII mutants and the evidence from other BMP and Activin signaling mutants and transgenics prompted us to further examine the role of canonical TGF-β signaling in anxiety-related behavior. Smad4 is the downstream common Smad required for all canonical BMP, Activin, and TGF-β signaling. In a previous report, we investigated the effect forebrain-specific loss of Smad4 (fbΔSmad4) on learning, memory, and anxiety, but only observed a modest increase in open-arm exploration in the EPM that was not significantly different than controls [18]. The responses of fbΔBMPRII mutants to light intensity prompted us to further examine anxiety-like behavior in fbΔSmad4 using the EPM. Follow-up experiments revealed that fbΔSmad4 mutants and control littermates would not participate (remained in the closed arm) at the same light intensity as used for fbΔBMPRII mutants and controls, likely due to genetic background differences. However, the fbΔSmad4 mice would initate arm exploration at approximately 50lux.

**Fig 3:**
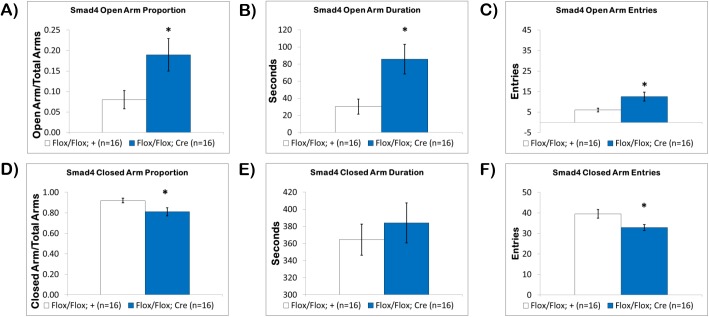
Loss of Smad4 Modulates Anxiety-Related Behavior in the Elevated Plus Maze. In the Elevated Plus Maze (A) the fbΔSmad4 mutant mice (blue) spent a significantly increased proportion of time exploring the open-arms, (B) had a significantly increased duration of time on the open-arms, (C) and made significantly more entries onto the open-arms compared to control littermates (white). When closed-arm exploration was examined, (D) the fbΔSmad4 mutant mice spent a significantly reduced proportion of time on the closed-arms, but (E) there was no significant difference in duration of time on the closed-arms, however, (F) the number of closed-arm entries was significantly reduced. Results reported as mean ± S.E.M. Asterisk indicates *p<0.05.

Under 50lux conditions, we discovered that naïve fbΔSmad4 mutants also spent significantly more time exploring the open-arms. The proportion of time spent on the open-arms **(Fig 3A)** for fbΔSmad4 mutants was 19.0± 4.0% compared to 8.0±2.2% for control littermates (p<0.05). The duration of time on the open-arms **(Fig 3B)** was also significantly increased, more than double, with fbΔSmad4 mutants spending 85.8±17.4 seconds compared to 30.3±8.8 seconds in control littermates (p<0.01). Open-arm entries were also significantly increased (p<0.01) with fbΔSmad4 mutants having 12.6±2.1 entries into the open-arms compared to 6.1±0.9 entries in control littermates **(Fig 3C)**.

When closed-arm time was examined, the proportion of closed-arm time and the number of closed-arm entries was significantly reduced in fbΔSmad4 mutants, but not the duration of closed-arm time. The proportion of time spent on the closed-arms **(Fig 3D)** in fbΔSmad4 mutants was 81.1±4.0% compared to 92.0±2.2% in control littermates (p<0.05). Although, the proportion of time was significantly reduced, the duration of time in the closed-arms **(Fig 3E)** was similar to controls with fbΔSmad4 mutants spending 384.0±23.5 seconds on the closed-arms compared to 364.0±18.2 seconds in control littermates. This discrepancy is due to the way that open and closed arm exploration is calculated (closed arm time/total arms vs. open-arms/total arms), as fbΔSmad4 mutants had more total arms time due to the significant increase in open-arm time. Lastly, the number of closed-arm entries was significantly reduced **(Fig 3F)** with fbΔSmad4 mutants having 32.9±1.4 entries compared to 39.5±2.1 entries in control littermates (p<0.05). These results show that canonical TGF-β signaling involving Smad4 also plays a role in anxiety-related behavior.

### The fbΔBMPRII Mutant Mice Show Increased Interest in Object Exploration

Although the EPM is generally used as an indicator of anxiety-related behavior, it also involves exploratory behavior. In order to further investigate exploratory behavior, a novel 4-cm diamater bottle cap was positioned in a corner of a 50x50x40cm (LWH) box, and naïve mice were allowed to explore freely for 15min. Exploratory behavior was analyzed by measuring object interaction, which included sniffing and climbing.

**Fig 4:**
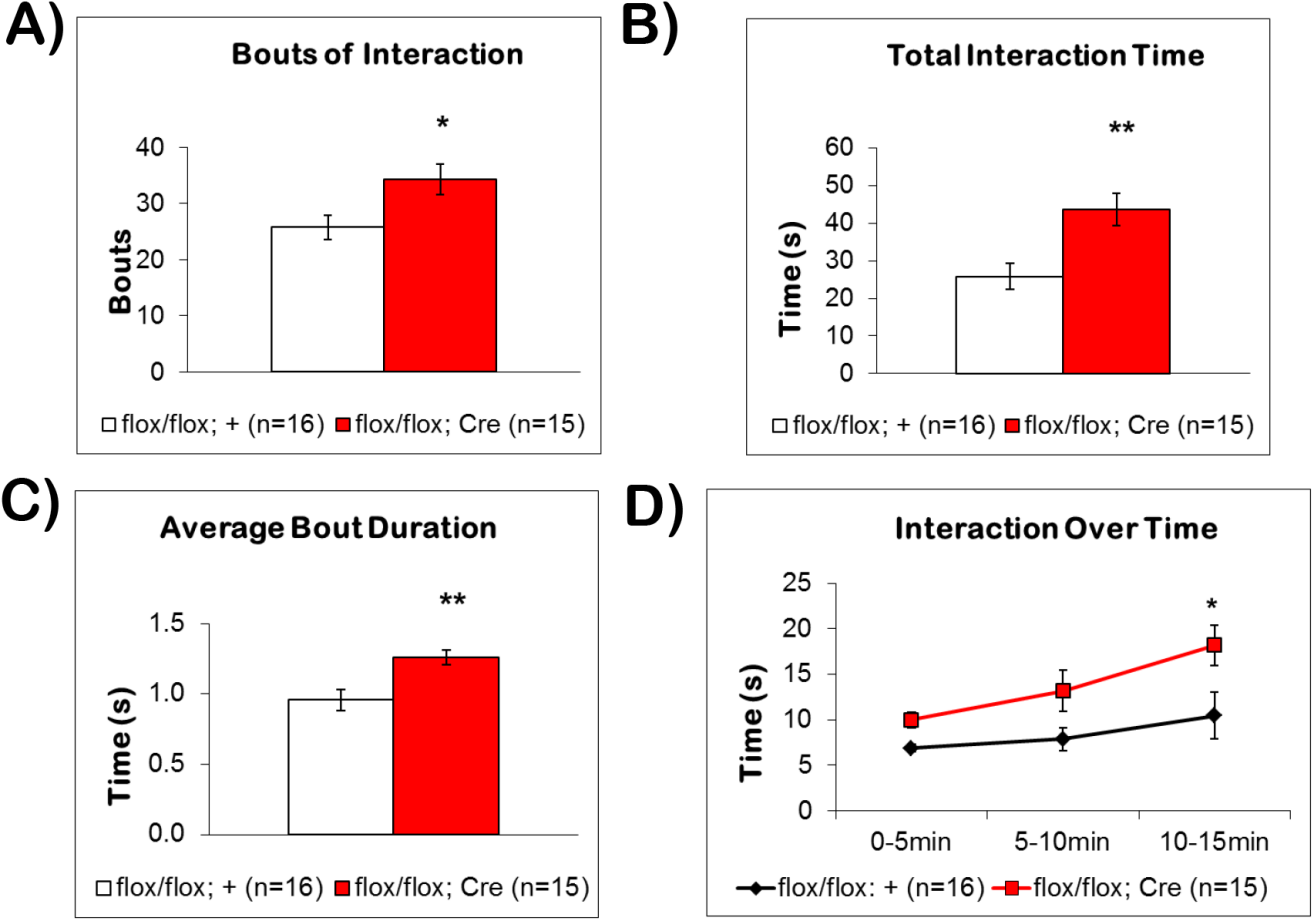
Loss of BMPRII Affects Object Exploratory Behavior. (A) The fbΔBMPRII mutant mice had significantly more bouts of exploring the novel object than control littermates. (B) When the total interaction time over the 15-minute trial was examined, fbΔBMPRII mutant mice spent significantly more time interacting with the novel object than control littermates. (C) When the duration of individual bouts was analyzed, fbΔBMPRII mutants had significantly longer bouts of interaction compared to control littermates. (D) When interaction behavior was analyzed over time in 5-minute bins, fbΔBMPRII mutants spent significantly more time exploring the novel object, compared to control littermates (F(1,29) = 8.91, p = 0.0057).Thus, fbΔBMPRII mutants show greater interest in object exploration than control littermates. Results reported as mean ± S.E.M. Asterisk indicates *p<0.05 or **p<0.005.

The fbΔBMPRII mutant mice displayed significantly more bouts of interaction (34 ± 3 bouts, p<0.05) compared to control littermates (26 ± 2 bouts) **(Fig 4A)**. Mutant mice also spent significantly more time **(Fig 4B)** sniffing and climbing on the novel object (43.6 ± 4.4s, p<0.005) compared to control littermates (25.8 ± 3.5s). We further analyzed the object interaction behavior by examining average bout duration **(Fig 4C)**. The fbΔBMPRII mutant mice exhibited significantly longer bout durations (1.26 ± 0.05s per bout, p<0.005) compared to control littermates (0.96 ± 0.08s per bout). When interaction behavior was examined in 5-min bins, we observed that fbΔBMPRII mutants spent more time exploring the novel object throughout the 15min of testing **(Fig 4D)**. A repeated measures two-way ANOVA revealed a significant effect of genotype (F(1,29) = 8.91, p = 0.0057), and time (F(2, 58) = 7.572, p = 0.0012). Post hoc analysis revealed that this difference was significant during the last 5 min (p<0.05). These results indicate that fbΔBMPRII mutants show significantly more interest in environmental exploration than control littermates.

### The fbΔBMPRII Mutants Show Normal Spatial Learning and Memory in the Morris Water Maze

The MWM is used to assess spatial learning and memory in mice [35–38]. In this task, mice must use distal cues to navigate a pool filled with opaque water in order to locate and remember the position of a hidden platform submerged below the water. In the reversal phase, the hidden platform is moved to a new position to test the ability of mice to extinguish the previous learning in order to learn the new position. As a control procedure, a visible cued platform is used the first day to screen for visual impairments and to teach the mice to search the pool for a platform **(S1 Fig)**. The MWM task is hippocampus-dependent, as lesions of the hippocampus are known to impair spatial learning ability [16].

The high expression of BMP signaling components in the hippocampus strongly suggests a role for BMP signaling in learning and memory. Studies of transgenic mice that over-express Noggin, a potent inhibitor of BMP ligands, using the neuron-specific enolase promoter (NSE-Noggin) show enhanced spatial learning and memory in a version of the MWM task in which the position of the hidden platform is changed daily. Conversely, transgenic mice that over-express BMP4 in the hippocampus and forebrain (NSE-BMP4) show impaired learning in this task [22]. Curiously, mice null for Chordin, another inhibitor of BMP ligands, initially show enhanced learning during the first hidden phase, but no improvements over controls thereafter [19]. Meanwhile, the fbΔSmad4 mutant mice, as well as BMPRIA and BMPRIB conditional double knockout mice, show normal spatial learning and memory in the MWM task [18,21].

In order to further investigate the role of BMP signaling in learning and memory, and the specific contributions of signaling through BMPRII, we tested fbΔBMPRII mice in the MWM.

**Fig 5:**
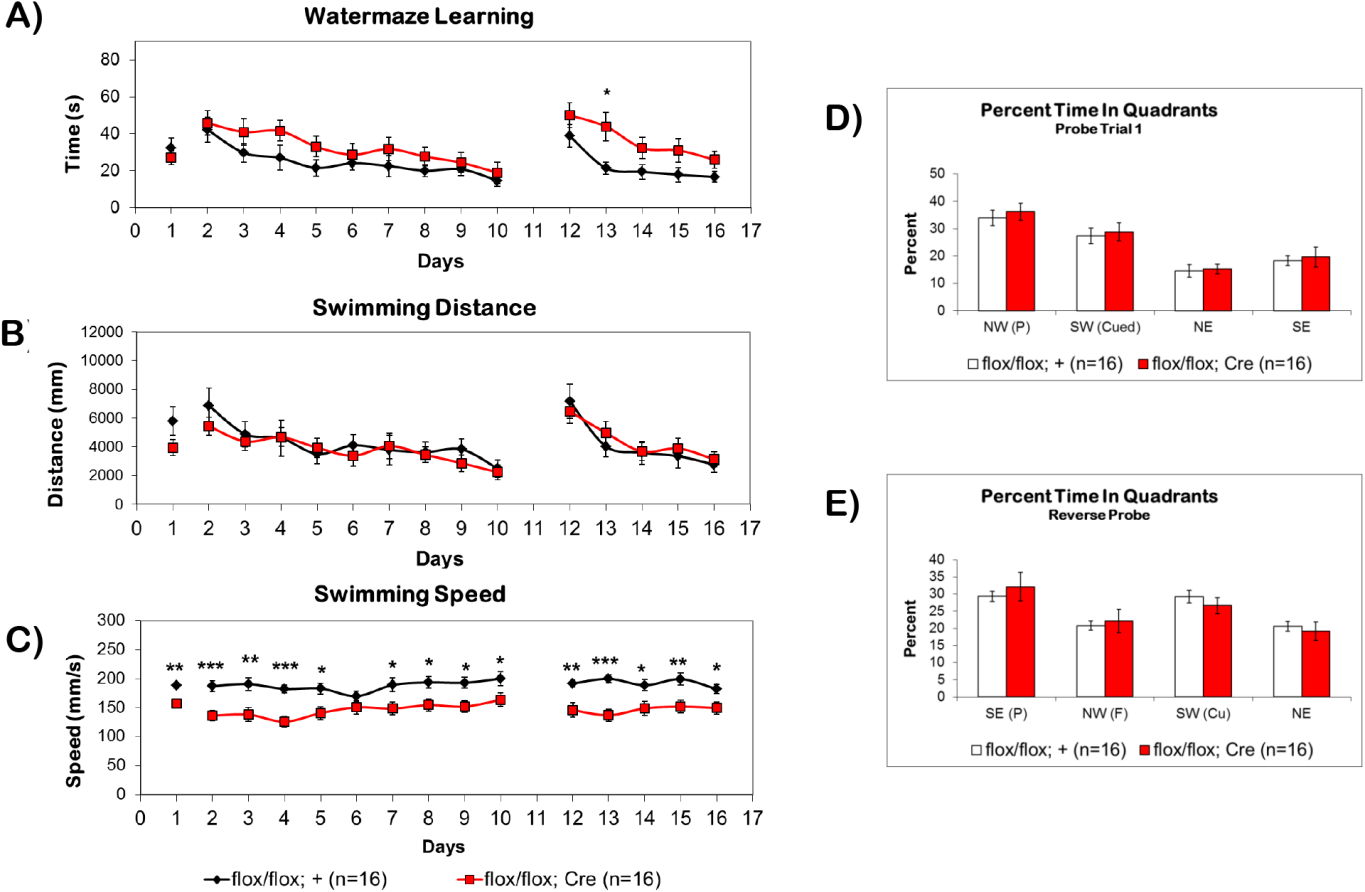
Spatial Learning and Memory in the MWM is Not Affected by Loss of BMPRII. (A) fbΔBMPRII mutant mice (red) do not have a significantly different learning curve than control littermates (black) during the cued (1) or first hidden (2–10) phases of the water maze, but show a slower learning curve during the reversal (12–16) phase of the water maze. (B) The swimming distance of the mice was not different than control littermates, (C) but the swimming speed of the mice was significantly slower on most days of the water maze. The appearance of a slower learning trend during the first hidden phase and reversal is likely an artifact of slow swimming speed rather than slower learning. (D-E) During probe trials fbΔBMPRII mutant mice show similar preference for the target quadrant as control littermates during both the first hidden phase and the reversal phase. Abbreviations: (P) platform position, (Cu) former cued platform position, (F) former hidden platform position. Results reported as mean ± S.E.M. Asterisk indicates *p<0.05, **p<0.005, ***p<0.0005, ****p<0.00005.

During the cued phase of the MWM on day 1, both fbΔBMPRII mutants and control littermates showed similar escape latencies. On days 2–10 of the first hidden platform phase, fbΔBMPRII mutants showed a tendency for slower escape latencies compared to control littermates, but there was no significant effect of genotype (F(1,30) = 3.044, p = 0.0913). During the reversal phase of the hidden platform, fbΔBMPRII mutants showed a slower escape latency that was much more pronounced than during the first hidden phase. A repeated measures two-way ANOVA revealed a significant effect of genotype (F(1,30) = 6.183, p = 0.0187). On the first day of the reversal, mutants and controls showed similar escape latencies, but on the second day of the reversal, fbΔBMPRII mutants took significantly longer to locate the hidden platform (p <0.05). On the remaining 3–5 days of the reversal, fbΔBMPRII mutants showed a tendency toward longer escape latencies to find the hidden platform, but once again they were not significantly different than controls **(Fig 5A)**.

We further analyzed data from the MWM test by examining swimming distances and speeds. We did not find any differences in swimming distances **(Fig 5B)** between fbΔBMPRII mutants and control littermates on any day of the first hidden phase (F(1,30) = 0.4272, p = 0.5183) or reversal phase of the MWM (F(1,30) = 0.1327, p = 0.7182). However, fbΔBMPRII mutant mice showed significantly slower swimming speeds compared to control littermates both during the first hidden phase (F(1,30) = 19.58, p = 0.0001) and the reversal phase (F(1,30) = 15.90, p = 0.0004). These slower swimming speeds impacted the escape latency curve **(Fig 5C)**, and the appearance of slower learning is likely an artifact of the slow swimming speed. Furthermore, the slow swimming does not appear to be a locomotor defect as fbΔBMPRII mutant mice did not show any defects in locomotion during the object exploration task, and actually had a trend of higher velocity and distance traveled than control littermates, but it was not statistically significant **(S2 Fig)**.

During probe trials, fbΔBMPRII mutant mice spent similar periods of time searching the platform quadrant as control littermates for both the first probe trial **(Fig 5D)** and the reverse probe trial **(Fig 5E)**, indicating that they learned the position of the platform as well as control littermates by the end of the training period. Overall, these results indicate that spatial learning and memory is intact in fbΔBMPRII mutants, and agrees with studies of BMPRIA/B conditional double knockouts, which suggest that BMP signaling is not required for spatial learning and memory.

### Forebrain Deletion of BMPRII Does Not Affect Immobility in the Porsolt Swim Test

**Fig 6:**
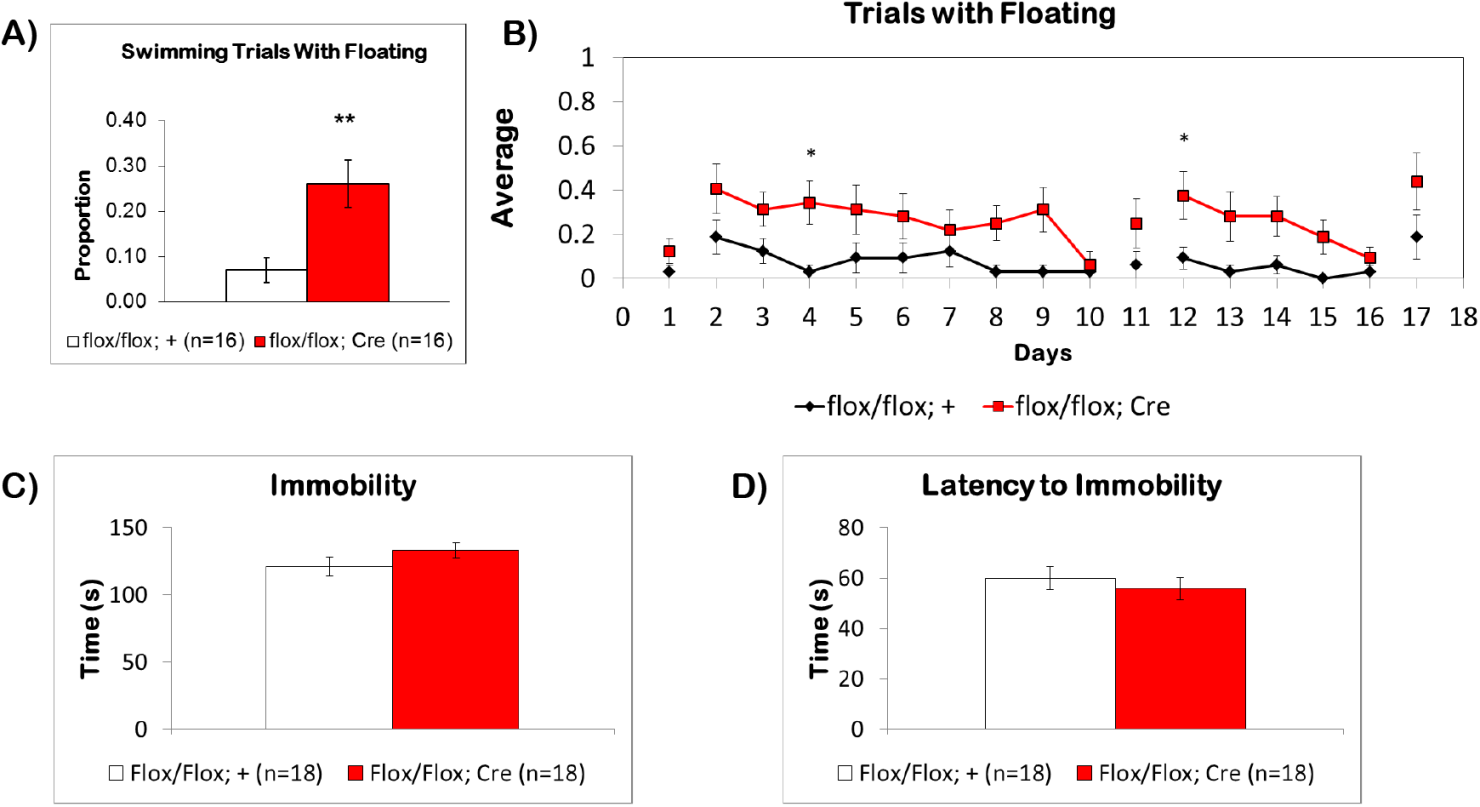
Floating Behavior in the Water Maze and Immobility of BMPRII Mice in the Porsolt Swim Test. (A) The fbΔBMPRII mutant mice (red) had significantly more trials with floating behavior during the water maze compared to control littermates, (B) and significantly more trials with floating across days, which impacted the average swimming speed of the mice, as well as the latency to find the hidden platform. (C) When tested in the PST, the fbΔBMPRII mutants did not have any differences in immobility compared to littermate controls, (D) and did not have any differences in the latency to first bout of immobility. Results reported as mean ± S.E.M. Asterisk indicates *p<0.05, **p<0.005.

During MWM training trials, we noticed that some of the mice spent time floating in the water rather than continuously swimming to find the platform. Floating here is defined as periods of time of at least 0.5 seconds where a mouse was immobile in the water without tail movement, appearance of leg paddling, and was simply drifting in the water by momentum. When we investigated this tendency, we found that fbΔBMPRII mutants exhibited significantly more floating behavior than control littermates (p < 0.005), with mutants floating in 26±5% of trials and control littermates in only 7±2% of trials **(Fig 6A)**. To further analyze this behavior, we averaged the number of trials with floating across days **(Fig 6B)**. This revealed a significant effect of genotype for both the first hidden (F(1,30) = 8.258, p = 0.0074) and reversal phases of the MWM (F(1,30) = 6.581, p = 0.0155). During the first hidden platform phase, floating behavior was significantly more than controls on day 4 and during the first day (day 12) of the reversal phase (p<0.05). In other behavioral paradigms, floating behavior is correlated with depressive-like behavior.

Due to the increased floating behavior observed in the MWM, we performed the PST, which is a standard procedure used to study learned helplessness, depression, or “behavioral despair” in rodents [39,40]. In this test, a single mouse is placed in a transparent water-filled container from which it cannot escape or touch the bottom, and is allowed to swim for a standard period of time. Depressive behavior is measured by analyzing swimming and time spent immobilized (floating). Increased immobility is associated with depressive-like behavior, since administration of anti-depressants reduces immobility and increases swimming.

Examination of the PST in naïve fbΔBMPRII mice did not reveal any differences in immobility **(Fig 6C)**. The average time spent immobilized by fbΔBMPRII mice was 133.2±5.7 seconds, while control littermates spent 121.1±6.8 seconds. There was also no significant difference in latency to the first bout of immobility **(Fig 6D)**. The latency to the first immobile period for fbΔBMPRII mice was 55.8±4.4 seconds, while the latency for control littermates was 59.9±4.5 seconds. These results indicate that in comparison to control littermates, fbΔBMPRII do not have any marked tendencies toward depressive-like behavior. It is possible, however, that the floating behavior observed in the MWM may be due to a stress-response not detected by this test.

### Forebrain Deletion of BMPRII Does Not Affect Acoustic Startle Reactivity or Prepulse Inhibition

Prepulse inhibition is a valid and reliable measure of sensorimotor gating and as such has been consistently employed in developing animal models for certain aspects of schizophrenia. Typically, PPI involves a reduction in the startle reflex response when a second stimulus of lower intensity is presented immediately prior to the startle noise stimulus.

Both mutant mice and littermate controls showed a normal startle stimulus intensity function, with higher dB levels producing larger startle responses. Although the mutant mice exhibited lower startle magnitudes compared to controls at the highest startle intensity (120db), this difference was not significant (t(29) = 1.53, p = 0.068). Similarly, there were no significant differences between groups in activity measurements while in the startle chambers (t(29) = 0.325, p = 0.75).

**Fig 7:**
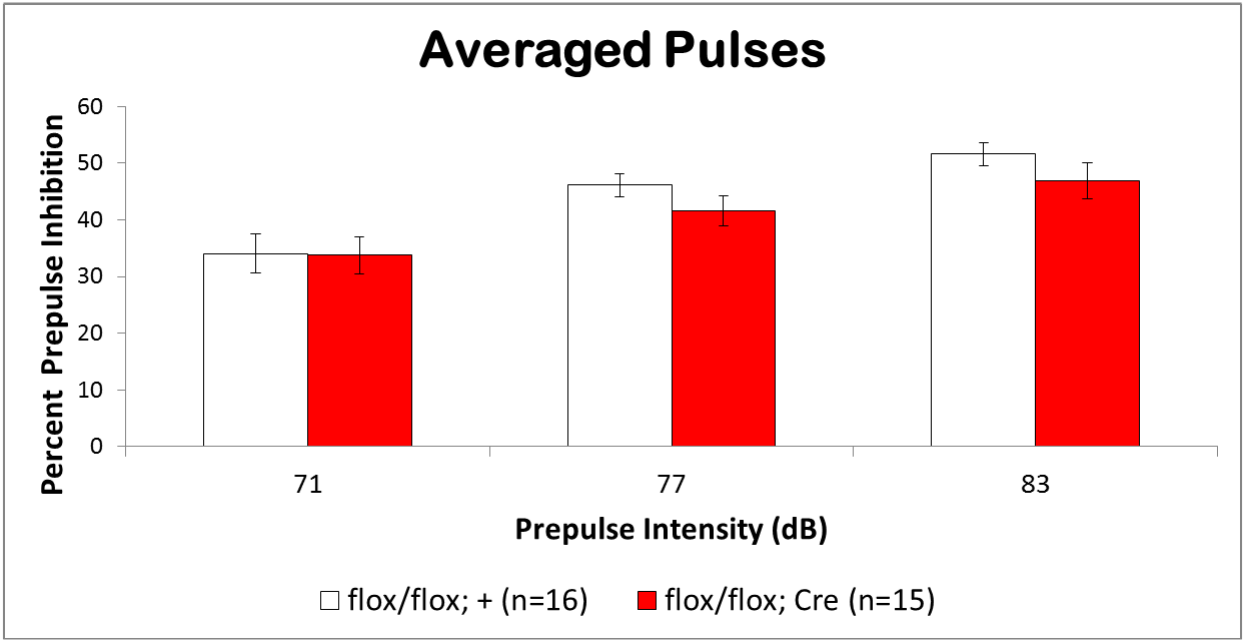
Prepulse Inhibition of the Acoustic Startle Reflex in fbΔBMPRII Mice. The fbΔBMPRII mutants (red) and control littermates (white) showed similar levels of prepulse inhibition of the acoustic startle across three different intensities of the prepulse stimulus. Thus, the mutant mice did not show deficits in sensorimotor gating.

Prepulse inihibition of the acoustic startle reflex (PPI) is a measure of sensorimotor gating, and impairments in PPI are associated with several psychiatric disorders, including schizophrenia [41]. Both the fbΔBMPRII mutant mice and littermate controls showed similar levels of PPI, which increased as a function of intensity of the prepulse stimulus **(Fig 7)**. A mixed factorial ANOVA (pulse x prepulse x group) revealed a significant effect of prepulse intensity levels (F(2, 276) = 15.02, p<0.0001), but no effects of group (F(1, 277) = 1.87, p = 0.17) or pulse level (F(2, 276) = 1.99, p = 0.14), and no other significant interactions (p>0.5). Since there were no interactions as a function of startle stimulus (pulse) intensity, Fig 7 depicts mean PPI across the three pulse intensities.

The absence of differences in mutant and control mice strongly suggests that fbΔBMPRII mutants have normal sensorimotor gating. Increased PPI as a function of prepulse intensity is consistent with previous findings.The absence of significant differences in startle amplitude and activity readings between fbΔBMPRII mutants and control mice indicates that hippocamups and forebrain deletion of BMPRII does not affect reflexive reactivity to a startle stimulus or locomotor activity in the confined space of the startle chamber.

## Discussion

Our studies indicate that fbΔBMPRII mutant mice show reduced anxiety-like behavior and increased exploratory behavior. Anxiety-like behavior has been previously described in mice in response to manipulations in TGF-β signaling pathways albeit with different paradigms and different genetic manipulations. For example, mice showed increased anxiety-like behavior in the open-field and light-dark box in experiments where Follistatin (a general inhibitor of both BMP and Activin ligands) was over-expressed in the hippocampus and forebrain using the CaMKIIα promoter, while mice that overexpress Activin using the CaMKIIα promoter show reduced anxiety-like behavior [23]. Additional experiments using inducible CaMKIIα-tTA>Follistatin transgenic mice have revealed deficits in fear conditioning, where Follistatin over-expressing mice freeze less during a foot-shock model of fear conditioning, while inducible over-expressing Activin mice showed increased freezing [42]. From these experiments, it is tempting to conclude that Activin signaling accounts for much of the modulation of anxiety-like behavior, but our experiments clearly indicate that BMPs also play a role since no Activin ligand has been shown to signal through BMPRII [43]. Consistent with a role for BMPs in modulating anxiety and fear, Gobeske et al. used the neuron-specific enolase (NSE) promoter, to over-express Noggin, a potent inhibitor of only BMP ligands, and found an increase in freezing during fear conditioning, while NSE-BMP4 over-expressing mice showed decreased freezing [22]. Although utilizing a different paradigm from that used here, these results are consistent with a possible role of BMPs in regulating anxiety and fear responses. However, there are other reports that confound simple explanations for the roles of Actvins and BMPs in anxiety-like responses. For instance, experiments in mice that express a dominant negative form of Activin receptor IB using CaMKIIα show reduced anxiety in the open-field, and in an elevated light-dark test [44]. Finally, mice with loss of both BMPRIA and BMPRIB in the forebrain using the Emx1 promoter also showed reduced anxiety in the elevated plus maze and defects in fear conditioning [21].

One possible explanation for some of the apparent inconsistencies is possible differences in the expression of promoters used to drive transgenic over-expression and Cre-recombinase. Although CamKIIα is reported to begin highest expression postnatally, both NSE and Emx1 begin expression during embryogenesis. Furthermore, although the hippocampus is thought to contribute to fear and anxiety behavior, it is of note that the promoters of CaMKIIα, Emx1, and NSE used in the different transgenic and conditional knockout mice are also expressed in the amygdala, which is classically associated with emotional and fear-related behavior [45–47]. Thus, subtle differences in the expression of Cre-recombinase in both the hippocampus and amygdala may account for some of the confounding results. In summary, despite some inconsistencies in the literature, the results in transgenic and knockout mice suggest, broadly speaking, that anxiety and fear-related behavior can be modulated through both canonical BMP- and Activin-mediated pathways. Future experiments using different combinations of receptor knockouts and a common Cre-recombinase line for all experiments should help elucidate the specific contributions of each signaling pathway to anxiety-related, fear-related, and exploratory behaviors.

Our studies also indicate that a reduction of BMP signaling in the hippocampus and forebrain by conditional deletion of BMPRII leaves spatial learning and memory intact, as well as sensorimotor gating in the acoustic startle response and PPI. Deficits in swimming speed were noted during the MWM due to a pronounced floating behavior, but the floating behavior was not due to behavioral-despair, as results of immobility in the PST were normal.

The lack of an effect on spatial learning and memory is not surprising as fbΔSmad4 mutant mice, as well as BMPRIA and BMPRIB conditional double knockout mice, also show normal learning and memory in the MWM task [18,21]. Curiously, Chordin null mice initially show enhanced learning during the first hidden phase, but no improvements over controls thereafter [19]. However, results in fbΔBMPRII mutant mice contrast with findings in NSE-Noggin mice, which show enhanced spatial learning and memory in a MWM task in which the position of the hidden platform is changed daily. NSE-BMP4 transgenic mice show impaired learning in this task [22]. One possible reason for this discrepancy is that over-expression of ligands and inhibitors can often produce more potent effects and phenotypes than single loss-of-function mutations due to receptor redundancy. Noggin is a potent inhibitor of all BMPs and leads to a complete loss of BMP signaling. In our system, loss of BMPRII causes a reduction of BMP signaling, but not a complete loss, as BMP ligands can still signal via BMPRIA/BMPRIB with ActRII or ActRII with ActRI(ALK2), all of which are expressed in the rodent hippocampus [6,7,9,11,21,48]. It is possible that this residual BMP signaling is sufficient to suppress the cognitive gains that are seen when BMP signaling is completely abolished through over-expression of Noggin. However, it would appear that loss of signaling through BMPRI or BMPRII or loss of canonical signaling through Smad4 generally does not disrupt spatial learning and memory.

Another intriguing possibility for the differences and similarities seen in the behavioral phenotypes of mutants and transgenics of BMP and Activin signaling may involve the neural progenitor cells (NPCs) of the subgranular zone (SGZ) of the hippocampus, as many of these studies report changes in the proliferation of these cells [21–23,49,50]. In fact, many of the anxiety-related, fear-related, learning and memory, and cognitive behavioral phenotypes appear to correlate better with the resultant changes in neurogenesis caused by the genetic manipulations rather than by the specific signaling pathways targeted. While studies of neurogenesis were beyond the scope of this study, future studies of adult neurogenesis in fbΔBMPRII mutants could help clarify whether the behavioral changes seen in this model are also correlated with this phenomenon. Lastly, future analysis of the roles of BMP, Activin, and TGF-β pathways in adult neurogenesis, learning, memory, exploratory behavior, and emotional behaviors, would benefit from employing the same Cre-recombinase drivers in side by side comparisons. Only in this way will it be possible to fully elucidate the unique contribution of each pathway to a particular behavior as well as the degree to which canonical versus non-canonical signaling impact the observations.

## Supporting Information

S1 Fig The Water Maze Scheme. (A) On day 1 a large, cued platform above water was placed in the SW quadrant. On days 2–10, a small, submerged platform was hidden below the water in the NW quadrant. On day 11 the platform was removed and mice were tested for their quadrant preference for 60s. On days 12–16 the small submerged platform was placed in the SE quadrant for the reversal. On day 17, the platform was removed and mice were tested for their quadrant preference for the reversal probe trial. The stars on the circle indicate the possible release positions used for the mice for each phase of the watermaze. (B) On each day mice were randomly assigned a release position. Mice were separated into groups of 8 mice (4 controls and 4 mutants). Positions were distributed such that each position was used equally on each day (see methods for more information). No mouse could swim from the same position twice in the same day.
(TIF)

S2 Fig Loss of BMPRII Does Not Impact Locomotion. During the object exploration task, fbΔBMPRII mutant mice did not have any defects in locomotion. There was a trend of (A) greater distance traveled and (B) higher velocity in fbΔBMPRII mutant mice compared to control littermates, but it was not statistically significant.
(TIF)
