# Supplementary figures and images for: Forebrain-Specific Loss of BMPRII in Mice Reduces Anxiety and Increases Object Exploration
Source: PLoS One. 2015 Oct 7;10(10):e0139860. doi: 10.1371/journal.pone.0139860 (PMC4596878; doi:10.1371/journal.pone.0139860)

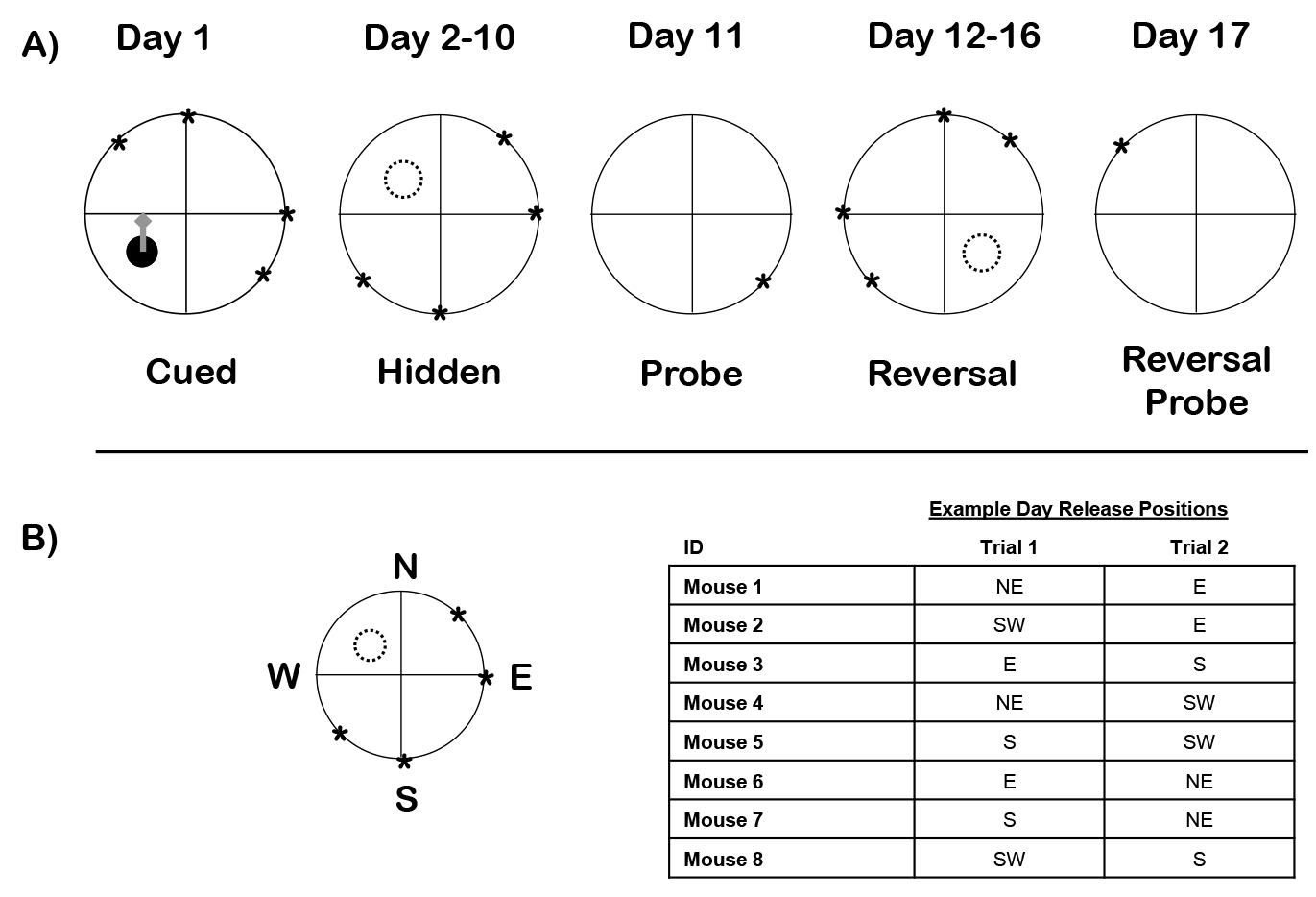

Supplement: S1 Fig — (A) On day 1 a large, cued platform above water was placed in the SW quadrant. On days 2–10, a small, submerged platform was hidden below the water in the NW quadrant. On day 11 the platform was removed and mice were tested for their quadrant preference for 60s. On days 12–16 the small submerged platform was placed in the SE quadrant for the reversal. On day 17, the platform was removed and mice were tested for their quadrant preference for the reversal probe trial. The stars on the circle indicate the possible release positions used for the mice for each phase of the watermaze. (B) On each day mice were randomly assigned a release position. Mice were separated into groups of 8 mice (4 controls and 4 mutants). Positions were distributed such that each position was used equally on each day (see methods for more information). No mouse could swim from the same position twice in the same day. (TIF) [file pone.0139860.s001.tif]

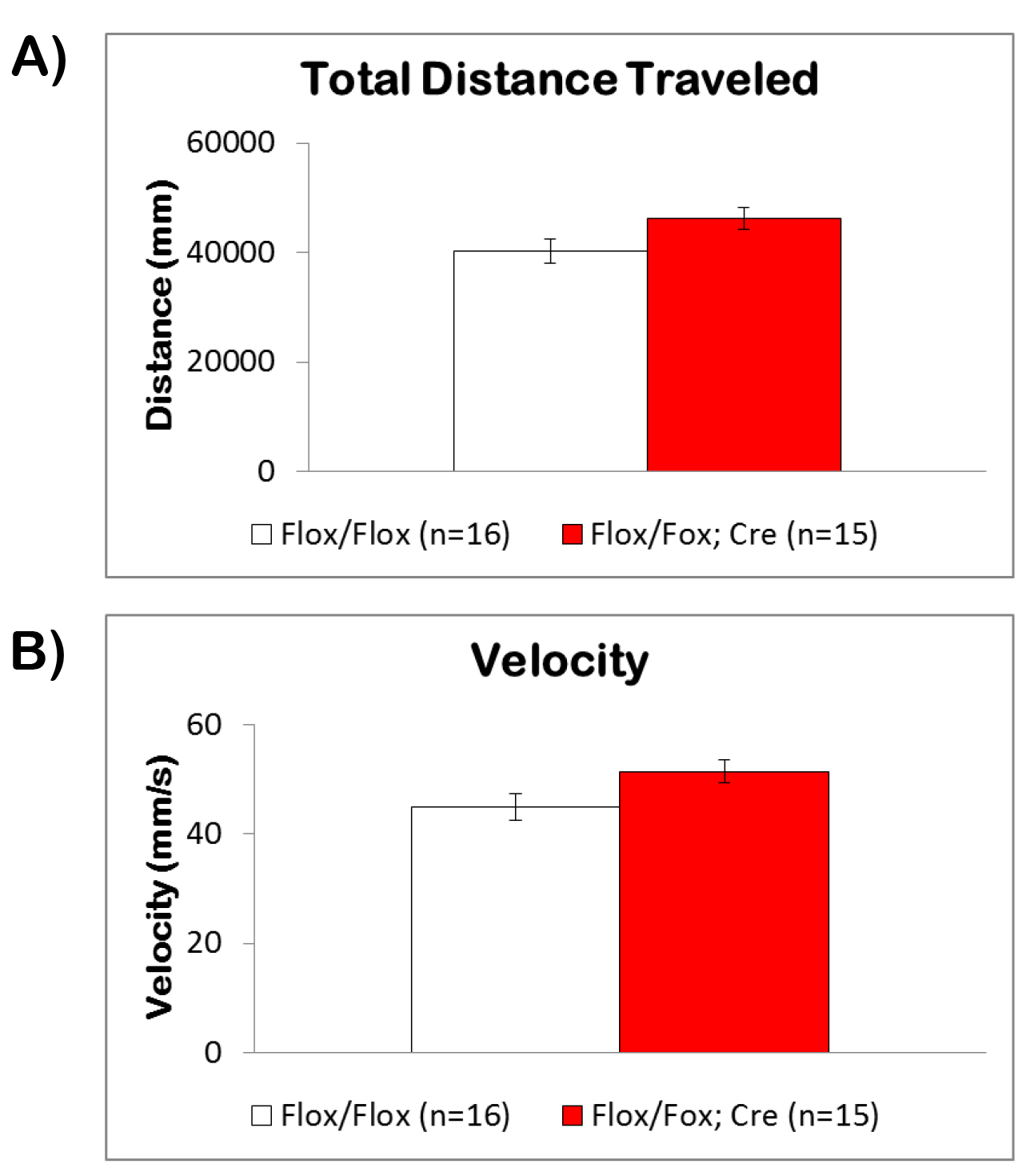

Supplement: S2 Fig — During the object exploration task, fbΔBMPRII mutant mice did not have any defects in locomotion. There was a trend of (A) greater distance traveled and (B) higher velocity in fbΔBMPRII mutant mice compared to control littermates, but it was not statistically significant. (TIF) [file pone.0139860.s002.tif]
